# Supplementary material for: Contribution of Adventitia-Derived Stem and Progenitor Cells to New Vessel Formation in Tumors
Source: Cells. 2021 Jul 7;10(7):1719. doi: 10.3390/cells10071719 (PMC8304670; doi:10.3390/cells10071719)
Supplement: Supplementary file 1 [file cells-10-01719-s001.zip › cells-1239814-supplementary.pdf]

Contribution of Adventitia-Derived Stem and Progenitor Cells to New Vessel Formation in Tumors

Berin Upcin [1], Erik Henke [1], Florian Kleefeldt [1], Helene Hoffmann [1], Andreas Rosenwald [2], Ster Irmak-Sav [3], Huseyin Bertal Aktas [4], Uwe Rückschloß [1] and Süleyman Ergün [1,*]

[1] Institute of Anatomy and Cell Biology, Julius-Maximilians-University, 97070 Würzburg, Germany; berin.upcin@uni-wuerzburg.de (B.U.); erik.henke@uni-wuerzburg.de (E.H.); florian.kleefeldt@uni-wuerzburg.de (F.K.); helene_hoffmann@gmx.net (H.H.); uwe.rueckschloss@uni-wuerzburg.de (U.R.)

[2] Institute of Pathology, Julius-Maximilians-University, 97070 Würzburg, Germany; rosenwald@uni-wuerzburg.de

[3] Faculty of Health Sciences, İstanbul Bilgi University, 34060 Istanbul, Turkey; ster.irmak@bilgi.edu.tr

[4] Department of Medicine, Hematology, Brigham and Women's Hospital, Boston, MA 02115, USA; huseyin_aktas@hms.harvard.edu

* Correspondence: sueleyman.erguen@uni-wuerzburg.de; Tel.: +49-931-31-82701

Supplementary Figures:

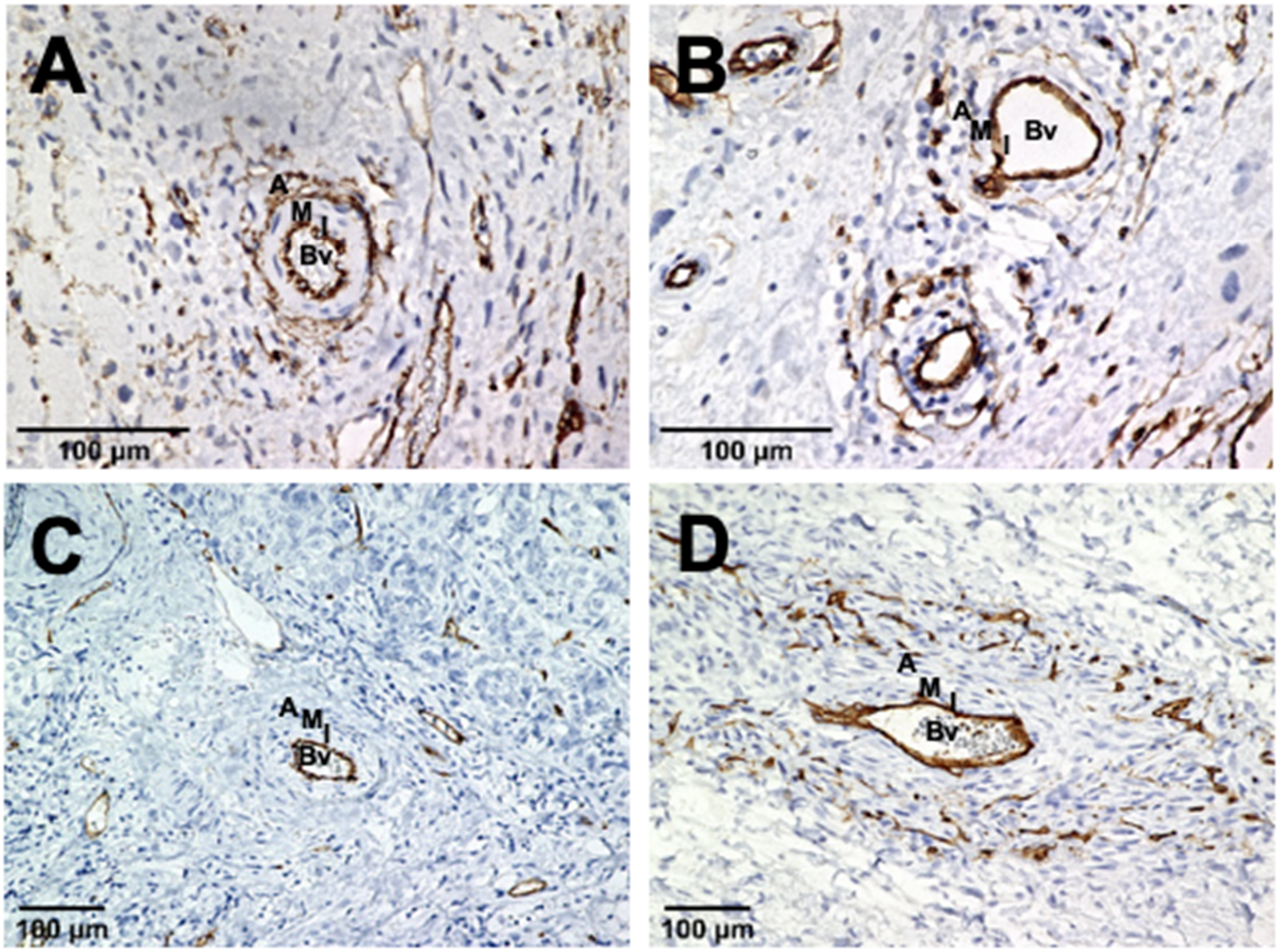

Figure S1. Mobilization of adventitia-resident CD34+ cells in human breast cancer. CD34+ cells in human breast cancer (A-D). CD34+ cells can be seen in the adventitia and partially formed like a capillary (A-B), the vessels of the tumor area no longer have any CD34+ cells in the adventitia (C-D). Abbreviations: Bv: Blood vessel: Tu: Tumor; I: vascular intima; M: vascular media; A: vascular adventitia.

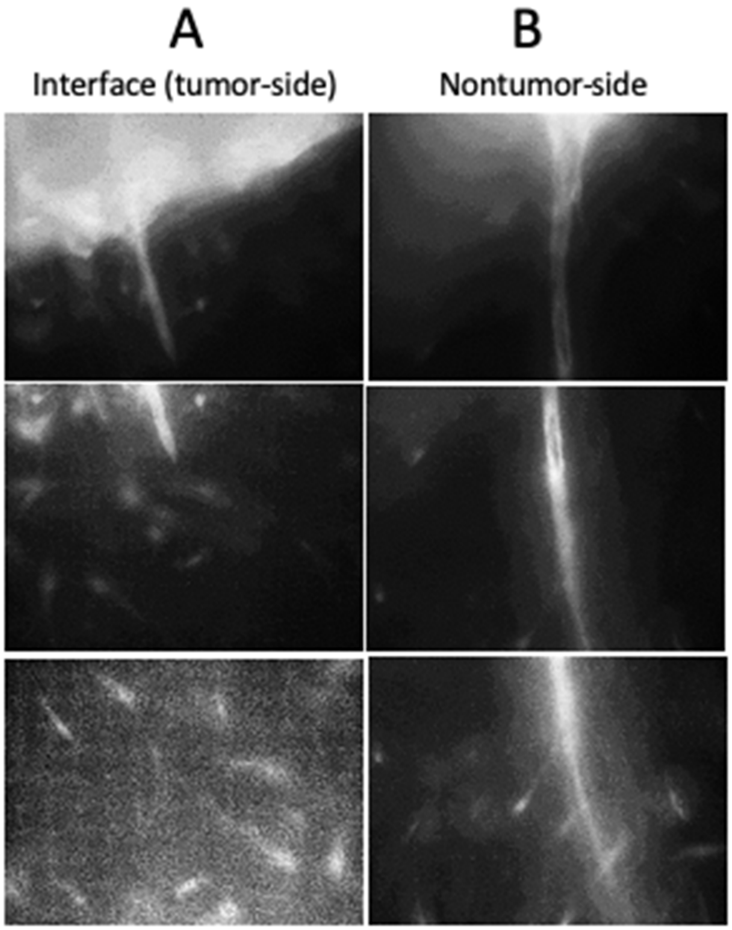

Figure S2. The length of capillary sprouts into the interface (tumor-side) and into the collagen gel at the non-tumor side of the Ars. The capillary sprouting was stopped at the interface (A), while an extended capillary sprouting was observed in the non-tumor side (B).

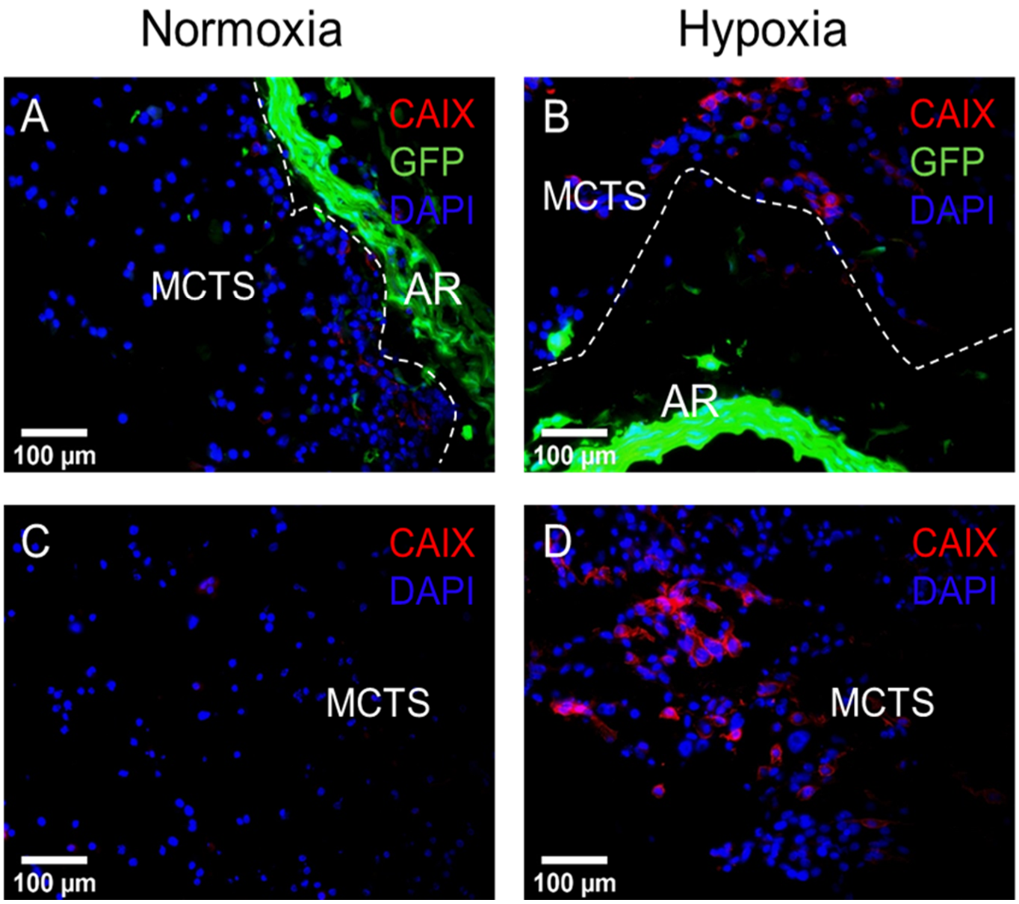

Figure S3. Increased CAIX expression in MCTS under hypoxia conditions. MCTS with AR were cultivated unter normoxia conditions for 14 days, had very less CAIX-expression (A-C), while CAIX was overexpressed by the MCTS which were cultivated under normoxia condition 3 and hypoxia conditions for 11 days (B-D).

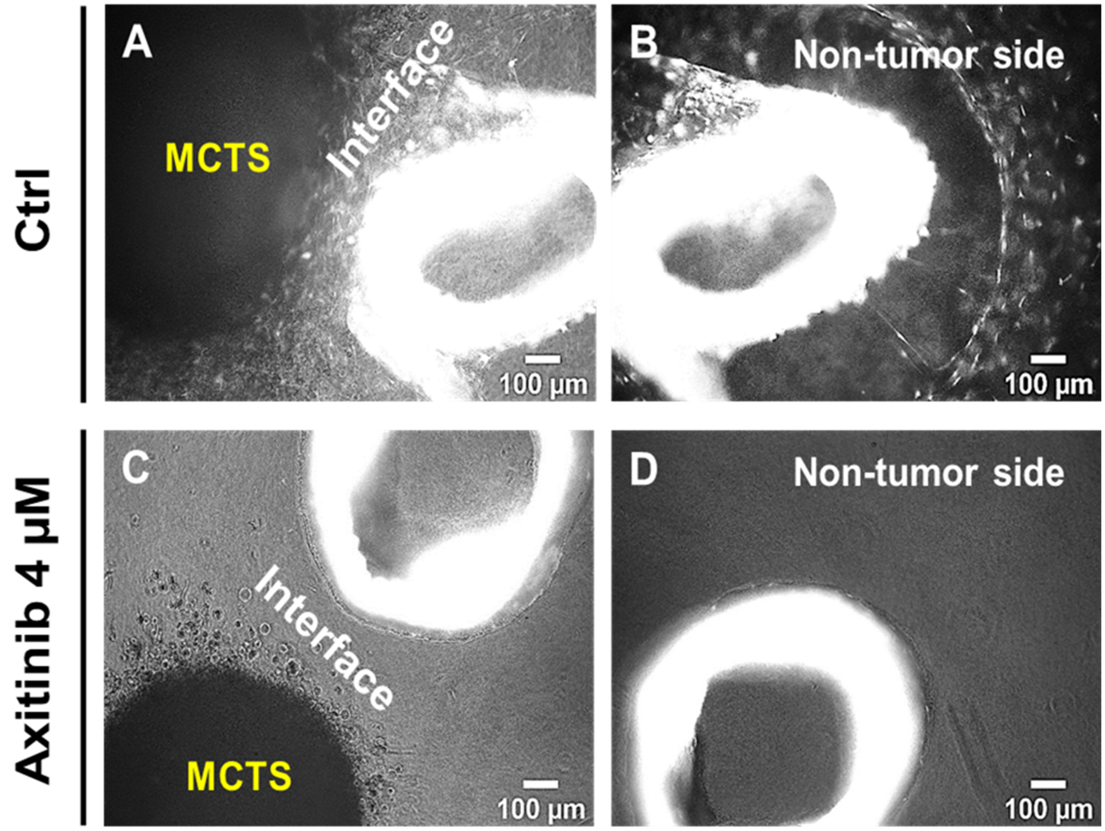

Figure S4. Inhibition of the sprouting by Axitinib. Sprouting of both ARs and MCTS was almost completely inhibited by axitinib (4μM) (C-D), while without axitinib treatment the sprouting was presence (A-B).
